# Application of Threshold Regression Analysis to Study the Impact of Clean Energy Development on China’s Carbon Productivity

**DOI:** 10.3390/ijerph17031060

**Published:** 2020-02-07

**Authors:** Dongri Han, Tuochen Li, Shaosong Feng, Ziyi Shi

**Affiliations:** School of Economics and Management, Harbin Engineering University, Harbin 150001, China; handongri@hrbeu.edu.cn (D.H.); sfeng.heu@gmail.com (S.F.); szy@hrbeu.edu.cn (Z.S.)

**Keywords:** clean energy development, technological innovation capability, carbon productivity, threshold model

## Abstract

Facing the pressures of international carbon emission reduction, the transformation into a low-carbon economy has become a common issue of all countries. The core of developing a low-carbon economy is to increase carbon productivity, which can be measured as the economic benefits of unit carbon emissions. Therefore, using province-level panel data in China from 2009 to 2017, we analyze the carbon productivity level of each region, and empirically investigate the threshold effect of clean energy development on carbon productivity under different technological innovation levels. The results show that the carbon productivity is rising, and China’s economic development pattern has been shifting towards low-carbon and sustainable development. Furthermore, the driving force of clean energy development on carbon productivity is not monotonously increasing (decreasing) but is a “double threshold effect” of technological innovation capability. Finally, based on the research conclusions and realistic requirements of China’s low-carbon economic transformation, this paper proposes improving carbon productivity from the aspects of innovation capability improvement and institutional guarantee.

## 1. Introduction

Due to the continuous accumulation of carbon dioxide and other greenhouse gas, a series of environmental problems, such as abnormal precipitation distribution, rising sea levels, and frequent floods and droughts, have brought serious threats to the living environment of human society. According to the report of the Second UN Environment Conference, about 7 million people worldwide die from pollution every year, of which 4.3 million are related to air pollution. Therefore, carbon emission control is an urgent issue of concern to countries around the world [1,2]. As the country with the largest carbon emission, China’s efforts are of great significance to the world’s carbon emission reduction process. However, as the largest developing country in the world, China faces serious regional development imbalances, and some underdeveloped areas have not yet escaped poverty [3]. Therefore, China must maintain economic growth while saving energy and reducing emissions [4]. Carbon productivity was first proposed by Kaya and Yokobori [5], which reflects the economic value of carbon dioxide as a factor input, that is the level of GDP output per unit of carbon dioxide. In 2008, McKinsey’s report “Carbon Productivity Challenges: Containing Global Change and Sustaining Economic Growth” clearly stated that any successful climate change mitigation technology must support the two goals of both stabilizing the greenhouse gas content in the atmosphere and maintaining economic growth, and the combination of the two goals is “carbon productivity”. China’s carbon productivity has excellent potential for improvement [6]. Therefore, improving carbon productivity is a realistic requirement for China to control greenhouse gas emissions, improve energy efficiency, balance economic growth, and achieve low-carbon development. Therefore, how to improve carbon productivity has become an important issue.

The burning of fossil energy generates a large amount of carbon dioxide, which constitutes about two-thirds of greenhouse gases, and causes great damage to the atmospheric environment. The new energy revolution advocates the use of wind energy, solar energy, and other clean energy sources to meet the growing human energy needs and gradually replace fossil energy, which is the fundamental means to control carbon emissions [7]. Therefore, increasing clean energy consumption and optimizing energy consumption structure are generally regarded as essential measures to promote the development of the low-carbon economy. Vigorously developing clean energy is not only an important measure to ensure energy security and control carbon dioxide emissions, but also plays a significant role in promoting the upgrading of industrial structure and carbon productivity. Considering the important role of clean energy and ensuring energy security, the Energy Law of the People’s Republic of China was promulgated, which encourages the development and utilization of clean energy, and defines solar energy, nuclear energy, marine energy, and other energy as clean energy [8]. However, it should be noted that, so far, no energy source can be called a completely clean energy source. For instance, although solar energy is a clean energy source because it does not emit environmentally harmful pollutants, nevertheless, from another aspect of analysis, an essential component of solar panels is silicon. When manufacturing solar panels, silicon needs to be melted and purified, which requires a lot of heat, which means that the atmospheric environment will be polluted during the process of manufacturing solar panels. Therefore, so-called clean energy is not an absolute concept [9].

Gross et al. [10] and Irandoust [11] noted that clean energy development tends to be technology-intensive. The large-scale development and low-cost use of clean energy require the support of an external technological innovation environment [12]. For instance, the construction of low-carbon cities requires the development and application of green technologies, including green buildings, smart lighting, and clean heating. At the same time, usually, the price of clean energy is high, and the market demand for clean energy by high-quality talents and high-tech companies is stronger. Therefore, in areas with high levels of technological innovation, the scientific research base and technology accumulation is relatively high, and infrastructure construction is complete. These regions have gathered a large number of high-quality talents and high-tech enterprises. This can not only improve the supply capacity of clean energy but also has enormous demand potential for clean energy. Therefore, based on the above analysis, and taking into account that under the Chinese context, the regional technological innovation capabilities are significantly different [13], this article considers that under the conditions of varying levels of technological innovation, the development space of clean energy is separate, and the role of improving carbon productivity may be different. It is necessary to divide the regional level of technological innovation according to a certain threshold, and then to find the optimal level of technological innovation that can promote the improvement of carbon productivity through a clean energy transition. To avoid the effects of empirical and randomness, this paper uses the nonlinear panel threshold regression model proposed by Hansen [14] to construct and clarify a nonlinear threshold model of clean energy development, technological innovation, and carbon productivity. This method identifies the data characteristics of unknown variables from the perspective of mathematical statistics, and determines the threshold value based on the principle of “the minimum of the squared residuals.” Furthermore, the error caused by artificially dividing the threshold variable interval is avoided, and the significance test of the endogenous threshold effect is scientifically and reliably performed [15].

We attempt to analyze the linkages between clean energy development, technological innovation, and carbon productivity by considering the perspective of the “threshold effect.” First of all, unlike previous studies that have used the number of patent applications or patent grants directly as a measure of technological innovation, we believe that technological development is a cumulative process, it should be considered as a concept of stock. Therefore, we convert the flow indicator of technology innovation capabilities into a stock indicator by using the perpetual inventory method. Secondly, due to the significant differences in technological innovation capabilities in different regions of China, we propose a hypothesis that there is a non-linear relationship between clean energy development and carbon productivity. Under the condition of technological innovation capabilities, the use of threshold models reveals how different levels of technological innovation capabilities affect the relationship between clean energy development and regional carbon productivity, and whether such a complicated relationship exists for thresholds. The research results can provide a useful reference for China’s regional energy transition and low-carbon economic growth.

## 2. Literature Review

Actively developing clean energy can not only meet the growing energy demand, reduce carbon dioxide emissions from fossil energy consumption, but also optimize economic structures and achieve sustainable economic growth. Hence, scholars are paying attention to the development of clean energy. The related research mainly involves two aspects, one is whether it can reduce carbon dioxide emissions, and the other is whether it can promote economic growth.

### 2.1. Can Clean Energy Development Promote Carbon Emission Reduction?

Through a study in the United States from 1960 to 2007, Menyah and Wolde-Rufael [16] proposed that the use of clean energy can promote carbon emission reduction. Similarly, Zafrilla et al. [17], through a carbon footprint analysis of Spanish nuclear facilities, found that nuclear power generation is an environmentally friendly clean technology that can effectively promote clean energy transition and reduce greenhouse gas emissions; at the same time, they proposed clean energy consumption will help Spain achieve its European energy roadmap goals. Panwar et al. [18] suggested that the over-reliance on fossil fuel consumption is the primary cause of climate change; they also believe that the use of wind energy, biogas technology, and biodiesel to promote clean energy has the potential to reduce greenhouse gas emissions. By comparing energy use in EU countries, Dogan and Seker [19] proposed that renewable energy consumption and trade liberalization can significantly contribute to carbon reduction. Similarly, Shafiei and Salim [20], Vasylieva et al. [21] all verified the conclusion that clean energy development has a carbon emission reduction effect.

In contrast, Apergis et al. [22] surveyed the energy use of 19 countries, based on the panel error correction model, noted that renewable energy is not significantly effective at reducing carbon dioxide emissions; mainly because of the shortage of storage technology, it is difficult to guarantee continuous supply effectively. Using the PVAR method, Kahia et al. [23] investigated the impact of renewable energy consumption on carbon dioxide emissions in the 24 Middle East and North Africa countries. The results showed that in economies with immature renewable energy sectors, the positive effect of renewable energy on carbon dioxide was not significant. Bölük and Mert [24] verified the carbon emission potential of renewable energy in Turkey, and the results showed that renewable energy could not effectively reduce carbon emissions in the short term. Similarly, Xu et al. [8] also pointed out that the development of clean energy is not always effective in controlling carbon emissions. In different stages of development, the impact of clean energy development on carbon dioxide emissions in the eastern, central, and western regions of China is disparate. Specifically, in the short term, clean energy development will not help reduce CO_2_ emissions in eastern China. Also, in the long run, the carbon emission reduction effect of clean energy development on the central and western regions cannot be reflected. Based on the results of the study, they suggested that the central and local governments should adopt timely measures based on the different roles of clean energy in various stages of development, to give full play to the catalytic role of clean energy development in reducing carbon dioxide emissions.

### 2.2. Can the Development of Clean Energy Promote Economic Growth?

The articles have three conclusions about the relationship between clean energy development and economic growth. The first is the promotion theory. For example, Lin and Li [25] pointed out that clean energy development can enhance the level of sustainable development and drive economic growth. By analyzing the benefits of investing in clean energy construction in Greece, Markaki et al. [9] proposed that investment in clean energy construction will increase GDP by 9.4 billion pounds per year and will provide more than 100,000 full-time jobs. The second is the inhibition theory. For instance, Qi and Li [26] pointed out that renewable energy consumption will cause economic costs. By using the autoregressive lag model to analyze energy growth in Norway and New Zealand, Fei et al. [27] proposed that using clean energy would cause some economic losses. Zhang and Liu [28] proposed that if China gradually increases its share of renewable energy to 35%, energy-driven price increases would depress consumption, investment, and output growth in the short term. By using a non-parametric additive regression model, Xu et al. [8] put forward that for China’s eastern and central regions, in the early stages of clean energy development, local governments need to invest a large amount of financial capital, supplemented by the necessary tax breaks and other preferential policies, to support clean energy development. Inevitably, this will increase the burden of regional economic growth, thus impeding economic growth. Thirdly, some scholars believe that there are regional differences in the impact of clean energy development on economic growth. For example, through empirical analysis of the energy structure and economic growth of the G20 countries, Sikder et al. [29] have suggested that renewable energy has different effects on the economic output of different countries; clean energy use can significantly boost gain in countries such as Argentina, Italy, and the US. Similarly, by comparing the fossil energy and non-fossil energy consumption in countries with an economic difference, Omri et al. [30] proposed that there is a one-way causal relationship between renewable energy consumption and economic growth in developed countries such as Japan and the Netherlands.

Scholars have conducted in-depth research on the role of clean energy development in economic growth and carbon dioxide emissions and affirmed the necessity of clean energy transformation. However, under the requirements of low-carbon development, the ultimate goal of factor allocation is not merely environmental protection or economic growth, but to achieve a “win–win” between them [31]. The question that arises is whether the high cost of clean energy development can be absorbed by the economic system in the short term and whether it can improve the level of sustainable development in the long run. In a developing country like China, for a long time to come, our primary development goal is still to promote economic growth, completely get rid of poverty, and achieve modernization. Therefore, exploring whether the development of clean energy can enhance carbon productivity and its intensity of action has essential reference value for China’s low-carbon economy transformation.

### 2.3. Carbon Productivity and Low Carbon Economy Development

Scholars have paid particular attention to the critical role of carbon productivity in the event of a low-carbon economy. For example, Mielnik and Goldemberg [32] proposed that carbon productivity is an essential criterion for the sustainability of economic development models in developing countries and is of great significance for achieving low-carbon economic development. Based on the concept of eco-efficiency, Kortelainen [33] used the Malmquist index to analyze the dynamic environmental performance of the EU 20 countries from 1990 to 2003 and reached similar conclusions. Using GMM-SYS estimation, Baldoni et al. [34] took the Italian farm group as an example to conduct an empirical study on the relationship between agricultural productivity levels and environmental performance. The research results confirmed that the link does exist, but the direction and degree of this relationship may differ significantly in different agricultural typologies. In China, scholars such as Zhan et al. [35] and He and Su [36] also pointed out that the key to China’s transformation of a low-carbon economy is to increase carbon productivity. On how to improve carbon productivity, Zhou et al. [37] pointed out that technological progress is a major cause of carbon emissions performance by measuring carbon emissions performance in 18 countries. Similar to this, Lin et al. [38] have confirmed the vital role of technological innovation in improving carbon productivity.

The literatures have provided rich and profound insights. In general, it can be expanded in the following two aspects. On the one hand, there are relatively few studies on clean energy development and carbon productivity, which are usually limited to carbon emissions, industrial upgrades, and economic development. This is not enough for China in the period of energy transition. It is even more necessary to explore the relationship between clean energy transition and low-carbon economic development based on the actual situation in various regions of China; that is, the effect of clean energy development on carbon productivity needs to be further discussed. On the other hand, previous studies have mostly studied the linear relationship between clean energy development and economic growth, technological innovation, or carbon productivity, and a large number of nonlinear relationships embodied in economic variables are ignored. There may be a threshold effect between the development of clean energy and carbon productivity, which is based on the threshold of technological innovation. Neglecting this nonlinear threshold will inevitably lead to biased results. Therefore, based on the technological innovation capabilities of 30 regions in China, this paper studies the relationship between clean energy development and carbon productivity. 

## 3. Methodology

### 3.1. Threshold Model Construction

Considering the differences in the level of technological innovation in various regions of China [39,40], there may be a threshold effect on the improvement of carbon productivity in clean energy development. It is necessary to divide the level of technological innovation according to a specific threshold value to accurately identify the direction and size of the role of clean energy development in carbon productivity. To avoid the random influence of artificial grouping, this paper chooses the nonlinear panel threshold model proposed by Hansen [14]. According to the variables selected in this paper, the threshold model is constructed as follows:

Single threshold model for clean energy development on carbon productivity:

(1) $$\begin{array}{cc} Productivity_{it} & =\theta +\alpha_{1}Gov_{it}+\alpha_{2}Human_{it}+\alpha_{3}Urban_{it}+\alpha_{4}FDI_{it}+\alpha_{5}IND_{it}\\ & +\beta_{1}Clean_{it}I\left(R\&D_{it}\leq \eta \right)+\beta_{2}Clean_{it}I(R\&D_{it}>\;\eta )+u_{i}+\varepsilon_{it} \end{array}$$

Double threshold model for clean energy development on carbon productivity:

(2) $$\begin{array}{cc} Productivity_{it} & =\theta +\alpha_{1}Gov_{it}+\alpha_{2}Human_{it}+\alpha_{3}Urban_{it}+\alpha_{4}FDI_{it}+\alpha_{5}IND_{it}\\ & +\beta_{1}Clean_{it}I\left(R\&D_{it}\leq \eta_{1}\right)\\ & +\beta_{2}Clean_{it}I\left(\eta_{1}<R\&D_{it}\leq \eta_{2}\right)+\beta_{3}Clean_{it}I\left(R\&D_{it}>\eta_{2}\right)+u_{i}+\varepsilon_{it} \end{array}$$

where i and t represent the province and year, respectively. For control variables, $Gov_{it}$ indicates government support, $Human_{it}$ indicates human capital, $Urban_{it}$ indicates urbanization level, $FDI_{it}$ indicates foreign trade dependence, $\;IND_{it}$ indicates industrial structure. For core variables, $Productivity_{it}$ indicates carbon productivity, $Clean_{it}$ indicates clean energy development, $R\&D_{it}$ indicates technological innovation capability. I(•) is an indicator function, $\eta_{1}$ is the single threshold value, $\eta_{2}$ is the double threshold value; $u_{i}$ is the individual fixed effect, $\varepsilon_{it}$ is a random interference term.

For the test of the nonlinear panel threshold model, firstly, each observation value is subtracted from the group average to eliminate the special effect, and the model’s dispersion form is obtained, and then the threshold value and parameters are jointly estimated. The rationality of the use of the threshold model depends on two tests: one is the existence test of the threshold effect, and the other is to verify the authenticity of the threshold estimate.

The null hypothesis of the existence test of the threshold effect is $H_{0}:\eta_{1}=\eta_{2}$, and the alternative hypothesis is $H_{1}:\eta_{1}\neq \eta_{2}$. The statistics built are

(3) $$F_{1}=\frac{SSE_{0}-SSE_{1}\left(\hat{\eta }\right)}{{\hat{\sigma }}^{2}}$$

where $SSE_{0}$ is the sum of squared residuals obtained by the model under the null hypothesis of the threshold existence test; since the threshold η is not recognized under the null hypothesis, the distribution of F statistic is non-standard. Hansen [14] obtained a uniform distribution of F statistic using the bootstrap method and acquired the probability value of rejecting the null hypothesis.

The null hypothesis that the estimated value of the threshold is equal to the actual cost is $H_{0}:\hat{\eta }=\eta_{0}$. The likelihood ratio statistic built is

(4) $$LR_{1}\left(\eta \right)=\frac{SSE_{1}\left(\eta \right)-SSE_{1}\left(\hat{\eta }\right)}{{\hat{\sigma }}^{2}}$$

Similarly, the distribution of LR statistic is also non-standard, and its asymptotic delivery satisfies $c\left(\tau \right)=-2\ln(1-\sqrt{1-\tau })$ under some specific assumptions. When $LR_{1}>c\left(\tau \right)$, the null hypothesis can be rejected, and the confidence interval of the threshold estimator can be obtained.

### 3.2. Variable Description and Data Processing

(1) Carbon productivity (Productivity). According to Kaya et al.’s definition [5], carbon productivity is the ratio of GDP of an economic entity to CO_2_ emissions over the same period, which represents the economic benefit of unit carbon emissions and is a core indicator for measuring the low carbon economy. For the calculation of CO_2_ emissions according to the calculation formula of IPCC (2006), based on energy consumption, this paper measures the carbon dioxide emissions of seven fossil energy sources such as coal and gasoline.
(2) Clean energy development (Clean). Due to the limitation of data availability, China’s National Bureau of Statistics does not make statistics on the production and consumption of clean energy. Therefore, this paper uses non-fossil energy consumption to indicate the degree of clean energy development in the region.
(3) Technological innovation capability (R&D). Since the number of patent applications is less affected by the efficiency and preference of patent agencies and directly reflects the level of technological innovation that is protected from external interferences, this paper uses the number of patent applications to build regional technological innovation capability indicators. Considering that technological innovation is a process of continuous accumulation, current technological innovation will have an impact on lagging carbon productivity. Therefore, unlike the existing literature, this paper uses the perpetual inventory method to process the inventory of patent applications to characterize the level of regional technological innovation.
  (5) $$R\&D_{i,t}=PAT_{i,t}+\left(1-\delta \right)R\&D_{i,t-1}$$
  where $R\&D_{i,t}$ denotes the technological innovation capacity stock in region i at year t. The stock of capacity is continually increased by new patents, captured by $PAT_{i,t}$. In addition, it is continuously replaced by new patents, obtained by a constant depreciation rate δ. Additionally, the initial value of the stock $R\&D_{i,t-1}$ is estimated as follows:
  (6) $$R\&D_{i,t_{0}}=\frac{PAT_{i,t_{0}}}{\left(\bar{g}+\delta \right)}$$
  where δ=0.1 is the depreciation rate according to the existing literature [41,42]; $\bar{g}$ is the average growth rate of patent count in the sample period.
(4) Government support (Gov). The government interferes with market operation through tax, loan, and capital control measures, which in turn affect the efficiency of resource allocation and the level of economic development. On the one hand, government support may make up for the lack of markets, provide support for advantageous industries, promote regional technological innovation capabilities and industrial upgrading, and thereby increase carbon productivity [43]. On the other hand, government support may also disrupt the market order, cause vicious competition, and reduce the efficiency of resource allocation, which is not conducive to regional sustainable development. This paper measures the extent of government support by the proportion of government expenditure to GDP; among them, government expenditure mainly includes expenditure on energy conservation and environmental protection, expenditure on ecological construction and environmental protection, investment in industrial pollution control, and expenditure on science and technology.
(5) Human capital (Human). Human capital is a clean production factor, providing more suitable production technology choices for enterprise development, supporting economic growth, and helping to alleviate the pressure of environmental pollution. Therefore, the dynamic accumulation of human capital has a sustainable growth effect and helps to increase urban carbon productivity. According to the definition of human capital, this paper uses the average years of education of the population over six years old in each region for the calculation. The education level of residents is divided into five categories. The formula for calculating the average number of years of education for residents is
  (7) Human=illiteracy∗0+primary∗6+junior∗9+senior∗12+college∗16
(6) Urbanization (Urban). The improvement of urbanization levels will create a large amount of infrastructure construction demand and real estate residential investment demand, which will lead to the development of related industries, such as cement, steel, and other energy-intensive industries. This will pose a considerable challenge to the improvement of the regional environment. This paper measures the level of urbanization by the proportion of the urban population to the total population.
(7) Foreign trade dependence (FDI). With the continuous strengthening of domestic and international trade links, regional energy cooperation and energy trade links have become increasingly close [44], affecting the energy security and green economy growth of various regions [3,45]. For one of the crucial ways of regional trade links, foreign direct investment, the larger the scale of foreign capital inflows, the stronger the effect of capital accumulation and technology spillovers, which may promote the green economic growth of capital inflows into the region. For another, foreign direct investment will inevitably bring about pollution shelter effects. This paper measures the dependence of international trade by the actual use of foreign investment in the proportion of GDP.
(8) Industrial structure (IND). The development of secondary industries is decisive for the economic growth of developing countries. At the same time, the development of the secondary sector will lead to a substantial increase in energy end-use, which will lead to a rise in carbon emissions. Therefore, the adjustment of industrial structures will have a meaningful impact on carbon productivity. This paper uses the ratio of the added value of tertiary industries to the added benefit of secondary industries to represent the industrial structure.

The data used in this paper are all from the China Statistical Yearbook, the China Environmental Statistics Yearbook, statistical yearbooks of various regions, and the National Energy Model Integration Platform of Beijing Institute of Technology. At the same time, we have processed the data necessary to control the effects of heteroscedasticity or estimation bias on accuracy. A descriptive statistical analysis of the sample is shown in Table 1.

## 4. Empirical Results and Analysis

### 4.1. The Analysis of Carbon Productivity

**Figure 1:**
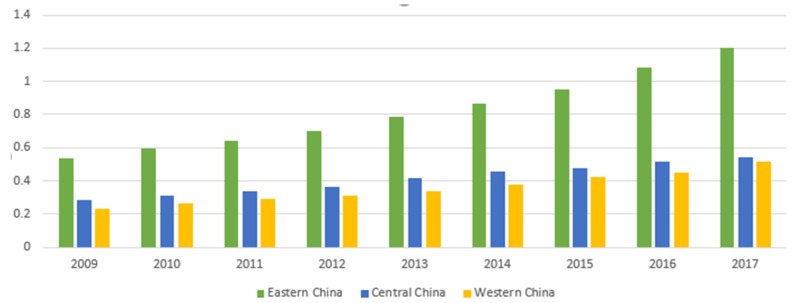
The carbon productivity data of China’s eastern, central, and western regions for 2009 to 2017.

After measuring the carbon emission data of each region, according to the definition of carbon productivity, this paper used the GDP data adjusted in 2009 as the base year to calculate the regional carbon productivity values. Considering the length of the article, this paper only lists the carbon productivity data of China’s eastern, central, and western areas for 2009 to 2017, as shown in Figure 1.

From 2009 to 2017, China’s carbon productivity increased yearly. The carbon productivity in the east of the region is significantly higher than in other areas, and the growth rate is impressive. The main reason is that the energy efficiency of the eastern part is substantially higher than in other regions. In 2017, the energy consumption per unit of GDP in the east of the region was 0.506 tons/10,000 yuan, while that in the central and western areas were 0.705 tons/10,000 yuan and 1.034 tons/10,000 yuan, respectively. At the same time, combined with carbon emissions and regional GDP data, it can be found that in 2017, the eastern region accounted for 38.8% of the country’s total carbon dioxide production while accounting for 57.9% of the country’s regional GDP, which shows that the overall economic intensification in the eastern region is higher. However, in the central area, carbon emissions were 40.5%, but the regional GDP accounted for only 26.4%, indicating that the economic development in the central and western regions still belonged to the extensive mode driven by factor input, with low economic output, high carbon emissions, and low carbon productivity.

In recent years, with the active support of relevant state policies and the efforts of the central and western regions, the regional economy has made significant progress, gradually entering the track of rapid development, and transforming into a low-carbon economy through the development of high-tech industries. For example, as the country’s first big data comprehensive pilot zone, Guizhou Province in the western region regards the development of the digital economy as a breakthrough for the catch-up to and a new engine for transformation and development; in 2017, it was the first to introduce a provincial digital economic development plan. Similarly, as a poverty-stricken province in the western region, Gansu Province has built a tens of kilowatt-class wind power base, and the construction of the station can save 250,000 tons of standard coal, carbon dioxide emissions of 427,000 tons, and reduce water consumption by 12,300 tons annually. These developments have a positive effect on improving the atmospheric environment. At the same time, they also promote employment and have excellent economic and social benefits.

### 4.2. Panel Threshold Model Results

Based on the panel threshold measurement method, this paper empirically investigates the complex mechanism between clean energy development and carbon productivity under the conditions of technological innovation capability in various regions. First, the threshold value of the F-value and the self-sampled p-value can be obtained by using technological innovation capability as the threshold value for the threshold effect test, as shown in Table 2 and Table 3. Referring to the Hansen threshold theory, the single and double threshold effects are tested; that is, there is a significant double threshold effect of technological innovation capability, and the threshold values are 12.4019 and 13.6268, respectively.

**Figure 2:**
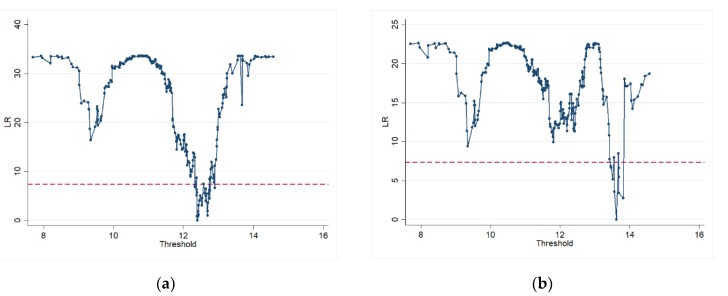
Estimation and confidence interval for the first threshold (**a**) and the second threshold (**b**) of energy misallocation.

After checking the threshold effect, the likelihood ratio function graph can be used to see the threshold value when the technological innovation capability is the threshold variable, that is, the 95% confidence interval. In the double threshold model studied in this paper, the estimated thresholds are 12.4019 (shown in Figure 2a) and 13.6268 (shown in Figure 2b), and the likelihood ratio statistic is zero. The 95% confidence interval for each threshold estimate is the interval formed by all the threshold values (corresponding to the dashed line in the figure) where the LR value is less than 5% of the significance level and is verified by the threshold truth value. Therefore, according to the two thresholds, China’s regions can be divided into three types: the low-tech innovation interval (R&D≤12.4019), the medium-tech innovation interval (12.4019<R&D≤13.6268), and the high-tech innovation interval (R&D>13.6268). 

The results of threshold regression (as shown in Table 4) show that the significantly facilitated clean energy in promoting carbon productivity improvement is limited by the threshold effect of technological innovation ability. When the level of technological innovation is low, the coefficient of influence of clean energy development on carbon productivity is −0.010, but the parameter estimates at this time do not pass the significance test. When the level of technological innovation crosses the first threshold, that is, technological innovation capability is between 12.4019 and 13.6268, clean energy development has a positive impact on carbon productivity, but is still not significant. As technological innovation capability continues to increase, the influence coefficient continues to grow. When the technological innovation capability crosses another threshold, namely 13.6268, the elastic factor of clean energy development to carbon productivity is 0.058 at a significant level of 5%, which shows that the high-tech innovation level range (R&D>13.6268) is a relatively optimal interval at which clean energy development can effectively increase carbon productivity.

With regards to the control variables, the elasticity coefficient of Gov for green development in various regions is 6.726 at the level of 1%, indicating that government support is a vital influence variable and is a strong driving force for promoting high-quality economic development. Therefore, the government should increase financial investment and technical support for the development of clean energy to promote the excellent development of the environmental protection industry. At the same time, while maintaining economic growth, the central government should take ecological benefit indicators as necessary evaluation indicators, which can prevent local governments from sacrificing the environment for GDP assessment. The elasticity coefficient of Human is 0.061 at the level of 5%, which indicates that human capital plays a role in promoting regional green development. Generally speaking, the development of clean energy often needs to be supported by necessary scientific research talents. Therefore, attention should be paid to the cultivation and introduction of high-end talents. The elasticity coefficient of FDI is 0.404 at the level of 5%, which indicates that the green technology brought by foreign investment significantly improves the green development level of regional enterprises, thus promoting economic growth and controlling carbon emissions. Therefore, the local government should further effectively attract foreign investment, but in the introduction of foreign investment, the local government will also need to prevent pollution problems. The effect of IND on carbon productivity is positive and significant, indicating that the regional industrial structure is one of the important variables affecting the green development of the region. This requires local governments to pay attention to the development of low-energy and high-tech industries, strengthen energy conservation and consumption reduction in the secondary sectors, and gradually reduce the proportion of high-energy sectors in China’s economic development. The effect of City on carbon productivity is positive but not significant, indicating that during the sample period, it did not have a considerable impact on the growth of carbon productivity.

### 4.3. Discussion

It can be seen from the empirical analysis that the effects of clean energy development on carbon productivity will be weakened or enhanced by regional characteristics, depending on the level of technological innovation in the region.

In the low-tech innovation level, clean energy development cannot effectively promote carbon productivity. The main reason is that, compared with fossil energy such as coal, the development of clean energy requires comprehensive technical support. The key to the large-scale development and utilization of clean energy is a technological breakthrough. Taking tidal energy as an example, although many areas in China contain a lot of tidal energy, simple single-bank two-way power generation can no longer meet the needs of today’s tidal power generation. How to solve the critical technologies of large-scale turbine construction and reduce operating costs becomes an essential problem.

In the middle and high technology innovation intervals, clean energy can promote the improvement of carbon productivity, and effectiveness of action increases with the level of technological innovation. The main reason is that the technology needed for the development of clean energy has been adequately supported, and clean energy can be rationally developed and used. These energy sources are mostly environmentally friendly energy sources with little pollution, which dramatically reduce greenhouse gas emissions such as carbon dioxide and increase carbon productivity. Besides, the improvement of technological innovation level can maximize the development and utilization of clean and efficient energy so that enterprises can abandon fossil energy, thereby promoting sustainable economic growth and increasing carbon productivity.

### 4.4. Time and Space Heterogeneity

**Figure 3:**
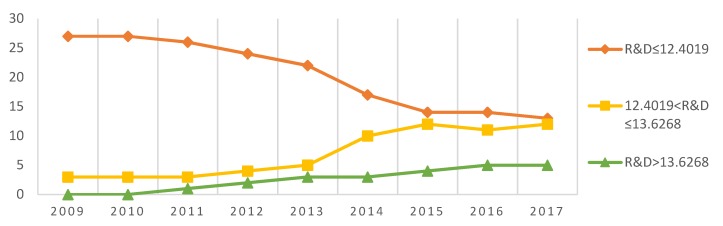
Technological innovation threshold level time trend.

According to the estimated values of the two threshold values: 12.4019 and 13.6268, the analysis of the relative threshold value distribution of technological innovation capability in 30 provinces of China between 2009 and 2017 can be further divided into three groups: the low-tech innovation capability interval (R&D≤12.4019), the medium-tech innovation capability interval (12.4019<R&D≤13.6268), and the high-tech innovation capability interval (R&D>13.6268); the results of the grouping are shown in Table 5. Further, Figure 3 shows the trend of the number of regions in different thresholds.

Overall, the sample size of the high-tech innovation capability in the relatively optimal range is small, e.g., 23 regions accounting for 10.9% of the total. There is a heterogeneity in the time and space of technological innovation capability in the process of clean energy development to promote carbon productivity improvement. The distribution of sample intervals in different years is quite different.

In terms of the time change, most provinces in China were in the low-tech innovation capacity range from 2009 to 2014. At this stage, most areas in China had poor technological innovation capabilities, which are not conducive to the driving effect of clean energy development and the promotion of carbon productivity. After 2015, China’s regional technological innovation capability significantly improved, and the number of medium- and high-tech innovation capability intervals increased dramatically, which actively promoted the development of clean energy and enhanced carbon productivity.

In terms of spatial change, according to the threshold level, during the sample period, only the eastern coastal regions of Beijing, Jiangsu, Zhejiang, Shandong, and Guangdong crossed the threshold and entered the high-tech innovation capability interval. These regions have not only higher technological innovation capabilities, but their economic development levels and human capital accumulation are also close to the level of developed countries. Therefore, the transformation of the low-carbon economy in these areas has achieved remarkable results, and the role of clean energy development in driving carbon productivity has been very significant. In the central and western regions, only seven provinces, including Anhui, Shanxi, and Henan, have entered the medium-tech innovation capability interval, and the overall technological innovation capability is relatively weak. Because these provinces are affected by factors such as resource endowment, talents, and the economic environment, the innovation base is soft, and industrial development is still driven by factor input. Some regions have not yet begun low-carbon transformation.

## 5. Robustness

To ensure the reliability of the results, we further conducted a robustness test to verify the impact of clean energy development on green economic growth under the threshold effect of technological innovation capacity. Improving energy efficiency is a significant way to ensure energy security and low-carbon economic development [46]. Therefore, in this paper, the Super-SBM model is used to calculate the total factor energy efficiency of 30 regions in China to represent the development status of the regional green economy.

From Table 6, we can see that when technological innovation capacity is lower than 13.8213, the development of clean energy cannot effectively promote the improvement of energy efficiency. When technological innovation capacity crosses the threshold, the role of clean energy development in improving energy efficiency can be realized. It indicates that the development of clean energy has a significant double threshold effect on energy efficiency and the research conclusion is very robust.

## 6. Conclusions

Based on the “new normal” background of economic transformation, this paper uses 30 sets of provincial-level panel data from 2009 to 2017 to construct a nonlinear threshold model that includes clean energy development, technological innovation capability, and carbon productivity. Combined with the spatial and temporal heterogeneity factors, the complex mechanism of clean energy development and carbon productivity is clarified. Through theoretical and empirical analysis, this paper has the following conclusions.

Clean energy development can increase carbon productivity. Facing the international community’s carbon emission reduction pressure, and based on the realistic requirements of China’s economic sustainability growth, the search for a sustainable development model that is suitable for economic growth and environmental protection has become an essential problem for the Chinese government. Promoting the energy production and consumption revolution and accelerating the transition of energy consumption to non-fossil energy are critical ways to ensure China’s energy security and steady development.

The effect of clean energy development on the promotion of carbon productivity is limited by regional technological innovation capability. As the technological innovation capability continues to cross the corresponding threshold, the contribution of clean energy development to carbon productivity has gradually changed from insignificant to significant, and the coefficient of action has changed from negative to positive, and there is a substantial double threshold effect. The results show that the improvement of regional technological innovation capability will provide sufficient technical support for clean energy development, and thus promote the low carbon transformation of the economy.

According to the threshold level, the area is divided into three types: a low-tech innovation capability interval (R&D≤12.4019), a medium-tech innovation capability interval (12.4019<R&D≤13.6268), and a high-tech innovation capability interval  (R&D>13.6268). In general, the level of technological innovation across China is quite different. Between 2009 and 2014, most areas, especially the central and western regions, were in the low-tech innovation capacity range. Since 2015, the situation has improved, but the sample size of the high-tech innovation capacity interval is still small. Besides, the technological innovation capacity of the central and western regions still has room for improvement.

Based on the research conclusions, to improve the ability of technological innovation and promote the positive effect of clean energy development on enhancing carbon productivity, we can work from the following aspects.

On the one hand, regional technological innovation capabilities must be improved. Whether clean energy can be used on a large scale is a question of the innovation of core technology. Although China is a big manufacturing country in the world, in terms of clean energy equipment manufacturing technology, there is still a big gap between China and developed countries such as the United States and Germany. If technical equipment cannot keep up, it will affect the industrialization of clean energy. This requires the government to provide sound policy support for technological innovation, and increase funding for clean technology innovation and actively guide social funds to invest in cleaner production. Attention should also be paid to strengthening cooperation with the international community, actively promoting the introduction of advanced technologies, and making technological breakthroughs based on technology import to improve the independent innovation capability of China’s low-carbon technologies. Also, we must pay attention to the cultivation of talents.

On the other hand, policy guarantees to promote the development of clean energy must be implemented. At this stage, China must first formulate relevant supporting policies, vigorously support and encourage the development and utilization of clean energy, continuously expand the proportion of clean energy in China’s energy structure, and expand the application of clean energy. Besides, government policies should be tilted towards environmental companies to reduce the financial pressure on companies to use clean energy and develop clean technologies, and to encourage them to rely on technology to promote the enthusiasm and initiative of clean energy development and green industrial transformation.

It should be pointed out that although some valuable research conclusions have been made in this article, there are still some limitations, which need to be further deepened in future research. In terms of research methods, a dynamic threshold model should be used to analyze the effect of economic green growth. For the research object, we should pay more attention to an industry’s specific effects and need to conduct a more targeted analysis for a certain industry.

## Figures and Tables

**Table 1:** Descriptive statistics of variables.

Variable	Mean	SD	Variance	Min	Max
Productivity	0.557	2.196	0.242	0.058	4.570
Clean	6.750	0.704	1.289	3.149	9.431
R&D	11.592	1.541	2.375	7.424	15.054
Gov	0.460	0.101	0.007	0.190	0.590
Human	9.075	1.735	1.056	6.764	13.525
City	0.553	0.649	0.017	0.299	0.896
FDI	0.103	1.772	0.032	0.008	1.102
IND	1.045	0.594	0.352	0.50	4.237

**Table 2:** Test results of threshold significance.

Threshold	*F*-Value	*p*-Value	Critical Value		
			1%	5%	10%
Single threshold	38.78 **	0.0400	93.7684	35.0672	29.3558
Double threshold	30.76 *	0.0800	55.3421	33.9254	26.5353
Triple threshold	12.71	0.6700	72.8003	57.1308	49.5696

**, * denote significant levels at 5%, and 10%, respectively.

**Table 3:** Threshold values and confidence intervals.

Model	Threshold Estimators	95% Confidence Intervals
Single threshold	12.4019	[12.3856, 12.4241]
Double threshold	13.6268	[13.5615, 13.6844]

**Table 4:** Estimation results of model parameters.

Productivity	Coef.	Std. Err	t	*p* > |t|	95% Conf. Interval	
Gov	6.726	0.632	10.64	0.000	5.480	7.971
Human	0.061	0.029	2.13	0.034	0.004	0.117
City	0.506	0.515	0.98	0.326	−0.508	1.521
FDI	0.404	0.155	2.60	0.010	0.098	0.709
IND	1.414	0.119	11.91	0.000	1.180	1.648
Clean (R&D≤12.4019)	−0.010	0.018	−0.55	0.583	−0.046	0.026
Clean (12.4019<R&D≤13.6268)	0.017	0.018	0.92	0.360	−0.019	0.053
Clean (R&D>13.6268)	0.058	0.019	3.03	0.003	0.020	0.096
cons	−4.907	0.474	−10.34	0.000	−5.842	−3.973

**Table 5:** Distribution of relative thresholds of technology innovation levels in 30 provinces of China from 2009 to 2017.

	12.4019<R&D≤13.6268		R&D>13.6268	
	Region	Number	Region	Number
2009	Jiangsu, Zhejiang, Guangdong	3		0
2010	Jiangsu, Zhejiang, Guangdong	3		0
2011	Zhejiang, Shandong, Guangdong	3	Jiangsu	1
2012	Beijing, Shanghai, Zhejiang, Shandong	4	Jiangsu, Guangdong	2
2013	Beijing, Shanghai, Anhui, Shandong, Sichuan	5	Jiangsu, Zhejiang, Guangdong	3
2014	Beijing, Tianjin, Shanghai, Anhui, Fujian, Shandong, Henan, Hubei, Sichuan, Shanxi	10	Jiangsu, Zhejiang, Guangdong	3
2015	Beijing, Tianjin, Liaoning, Shanghai, Anhui, Fujian, Henan, Hubei, Hunan, Chongqing, Sichuan, Shanxi	12	Jiangsu, Zhejiang, Shandong, Guangdong	4
2016	Tianjin, Liaoning, Shanghai, Anhui, Fujian, Henan, Hubei, Hunan, Chongqing, Sichuan, Shanxi	11	Beijing, Jiangsu, Zhejiang, Shandong, Guangdong	5
2017	Tianjin, Hebei, Liaoning, Shanghai, Anhui, Fujian, Henan, Hubei, Hunan, Chongqing, Sichuan, Shanxi	12	Beijing, Jiangsu, Zhejiang, Shandong, Guangdong	5

**Table 6:** Results of the robustness test.

Productivity	Coef.	Std. Err	t	*p* > |t|	95% Conf. Interval	
Gov	−0.100	0.186	−0.53	0.593	−0.466	0.267
Human	0.024	0.008	2.78	0.006	0.007	0.040
City	0.242	0.146	1.66	0.098	−0.045	0.530
FDI	0.164	0.045	3.65	0.000	0.076	0.253
IND	−0.082	0.035	−2.33	0.021	−0.151	−0.013
Clean(R&D≤13.1267)	−0.004	0.005	−0.78	0.436	−0.015	0.006
Clean(13.1267<R&D≤13.8213)	0.011	0.005	2.07	0.139	0.001	0.022
Clean(R&D>13.8213)	0.027	0.006	4.73	0.000	0.015	0.038
cons	0.300	0.139	2.15	0.032	0.026	0.575
